# Supplementary material for: Integrated Lipidomics and Transcriptomics Analyses Reveal Key Regulators of Fat Deposition in Different Adipose Tissues of Geese (Anser cygnoides)
Source: Animals (Basel). 2024 Jul 5;14(13):1990. doi: 10.3390/ani14131990 (PMC11240315; doi:10.3390/ani14131990)
Supplement: Supplementary file 1 [file animals-14-01990-s001.zip › Table S1.pdf]

Table S1. Ingredient and nutrient levels of the diet

Item	1-28d	29-300d
Ingredient %		
Corn	64.0	61.5
Soybean meal (Crude protein, 44%)	27.0	13.6
Fish meal (Crude protein, 56%)	3.0	3.0
Alfalfa meal (Crude protein, 16%)	2.0	16.0
Soybean oil	0.0	2.0
Dicalcium phosphate	1.8	1.9
Limestone	0.9	0.8
Salt	0.3	0.2
Premix[1]	1.0	1.0
Nutrient levels[2]		
Metabolic energy, KJ/kg	11.20	10.85
Crude protein, %	19.86	15.75
Ca, %	0.80	0.80
Total phosphorus, %	0.42	0.37
Crude fiber, %	4.85	6.00
Lys, %	0.90	0.65
Met, %	0.40	0.33
Sulphur-amino acid, %	0.79	0.56
Trp, %	0.17	0.13
Thr, %	0.80	0.80
Na, %	0.30	0.30
Cl, %	0.25	0.25

Note: [1] Vitamin and mineral premix supplied kilogram diet: vitamin A, 20,000 IU; vitamin D3, 4,500 IU; vitamin E, 300 IU; vitamin K3, 20 mg; vitamin B1, 10 mg; vitamin B2, 120 mg; vitamin B6, 20 mg; vitamin B12, 0.2 mg; nicotinic acid, 600 mg; pantothenic acid, 180 mg; folic acid, 10 mg; folate, 10 mg; biotin, 0.8 mg; choline, 7 g; Fe, 1.2 g; Cu, 0.2 g; Mn, 1.9 g; Zn, 1.8 g; I, 10 mg, Se, 6 mg.

[2] Analyzed values.
