# Supplementary figures and images for: Integrated Lipidomics and Transcriptomics Analyses Reveal Key Regulators of Fat Deposition in Different Adipose Tissues of Geese (Anser cygnoides)
Source: Animals (Basel). 2024 Jul 5;14(13):1990. doi: 10.3390/ani14131990 (PMC11240315; doi:10.3390/ani14131990)

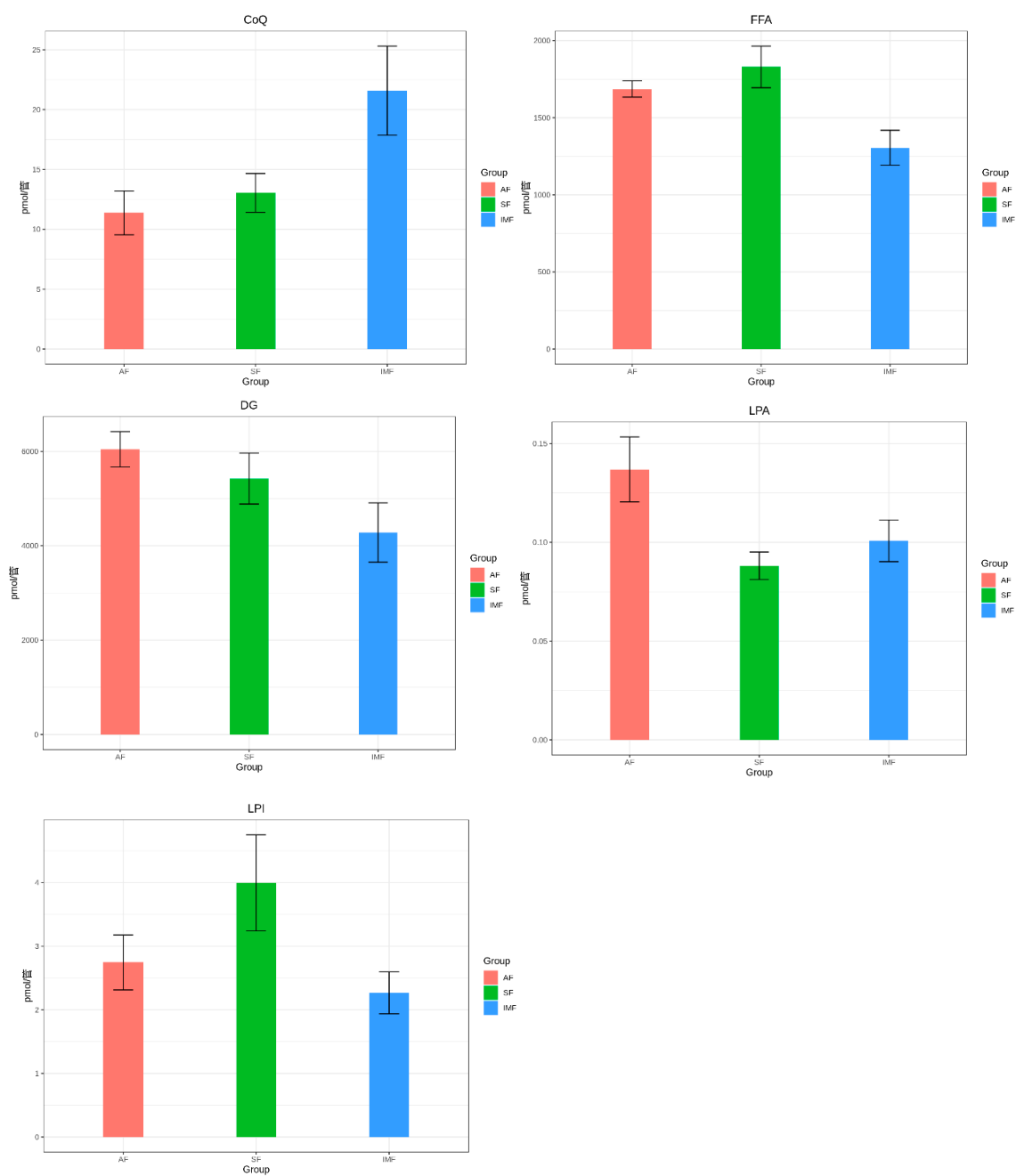

**Figure S1** Comparison of subclass content of lipid molecules

Supplement: Supplementary file 1 [file animals-14-01990-s001.zip › Figure S1.pdf]
